# NUP98 oncofusions in myeloid malignancies: An update on molecular mechanisms and therapeutic opportunities

**DOI:** 10.1002/hem3.70013

**Published:** 2024-09-25

**Authors:** Milad Rasouli, Selina Troester, Florian Grebien, Bianca F. Goemans, C. Michel Zwaan, Olaf Heidenreich

**Affiliations:** ^1^ Princess Maxima Center for Pediatric Oncology Utrecht The Netherlands; ^2^ Department of Pediatric Hematology/Oncology Erasmus MC‐Sophia Children's Hospital Rotterdam The Netherlands; ^3^ Department of Biological Sciences and Pathobiology University of Veterinary Medicine Vienna Vienna Austria; ^4^ St. Anna Children's Cancer Research Institute (CCRI) Vienna Austria; ^5^ CeMM Research Center for Molecular Medicine of the Austrian Academy of Sciences Vienna Austria; ^6^ Department of Hematology University Medical Center Utrecht Utrecht The Netherlands; ^7^ Wolfson Childhood Cancer Research Centre, Translational and Clinical Research Institute Newcastle University Newcastle upon Tyne UK

## Abstract

Acute myeloid leukemia (AML) is an aggressive hematological malignancy with a heterogeneous molecular landscape. In the pediatric context, the *NUP98* gene is a frequent target of chromosomal rearrangements that are linked to poor prognosis and unfavorable treatment outcomes in different AML subtypes. The translocations fuse *NUP98* to a diverse array of partner genes, resulting in fusion proteins with novel functions. NUP98 fusion oncoproteins induce aberrant biomolecular condensation, abnormal gene expression programs, and re‐wired protein interactions which ultimately cause alterations in the cell cycle and changes in cellular structures, all of which contribute to leukemia development. The extent of these effects is steered by the functional domains of the fusion partners and the influence of concomitant somatic mutations. In this review, we discuss the complex characteristics of NUP98 fusion proteins and potential novel therapeutic approaches for NUP98 fusion‐driven AML.

## INTRODUCTION

Leukemia is the most common childhood cancer, with acute lymphoblastic leukemia (ALL) and acute myeloid leukemia (AML) accounting for 80% and 15%–20% of all cases, respectively. Overall, pediatric AML has a long‐term survival rate of 60%–80%; however, for cytogenetic high‐risk subgroups survival rates drop to 30% and less.^1^, ^2^, ^3^, ^4^, ^5^ AML is a genetically heterogeneous disorder, that is characterized by uncontrolled clonal growth of immature hematopoietic cells with impaired differentiation. Genetic alterations can lead to the formation of chimeric proteins that play a critical role in the pathophysiology of leukemia. The genes involved in these rearrangements can encode transcription factors, epigenetic writers that possess histone posttranslational modification (PTM) activity, or epigenetic readers, which may recruit effector proteins to aberrant genomic sites. Histone PTMs are important determinants of gene regulation, and many oncogenic fusion proteins in AML can write, erase, or read activating or repressive histone marks, thus utilizing this mechanism to shape their own distinct transcriptional programs and oncogenic changes. Some chimeric fusion proteins can also interact with and recruit other regulatory proteins to alter the expression of hematopoiesis‐related genes.^6^, ^7^

Recurrent chromosomal rearrangements involving the *Nucleoporin 98* (*NUP98*) gene are observed in 5%−10% of pediatric AML cases and in approximately 2%–4% of adult AML cases, categorizing it as a high‐risk subtype in both childhood and adult leukemias.^8^, ^9^, ^10^, ^11^, ^12^, ^13^, ^14^, ^15^, ^16^, ^17^ Children and young adults with this rearrangement show a complete remission rate of 50% after one course of induction therapy, with overall poor survival rates of 25%–35%, and they face a substantially high risk of disease relapse, which ranges from 64% to 68%.^11^, ^18^ *NUP98::NSD1* and *NUP98::KDM5A* represent the most prevalent fusion events.^18^, ^19^ Specifically, *NUP98::NSD1* is detected in 8% in children and young adults with AML cases, marking a substantial occurrence within this group. Although the *NUP98::KDMA5* fusion is less frequent, occurring in only 1.4% of pediatric AML cases, its relevance increases in specific subtypes, such as acute megakaryoblastic leukemia (AMKL), where it is present in 10% of patients.^11^, ^18^, ^19^ *NUP98* fusions are predominantly linked to myeloid malignancies such as AML, chronic myeloid leukemia in blast crisis and mixed phenotype acute leukemia.^18^, ^20^, ^21^, ^22^, ^23^, ^24^, ^25^ Although rare in B‐cell malignancies, about 12% of patients with NUP98 fusions are associated with T‐cell acute lymphoblastic leukemia (T‐ALL).^22^ Since the presence of *NUP98* fusions in leukemia patients is associated with high induction failure and a low survival rate, understanding the molecular landscape of this leukemia subtype is critical for improving therapeutic options for this group of AMLs.

Herein we review the physiological and pathological molecular mechanisms of wild‐type and fusion forms of the NUP98 protein. Furthermore, we discuss the genomic landscape of *NUP98* fusion‐driven leukemia and highlight prospective treatment options for this AML subtype.

## ROLES OF WILD‐TYPE NUP98 IN NORMAL CELL PHYSIOLOGY

**Figure 1:**
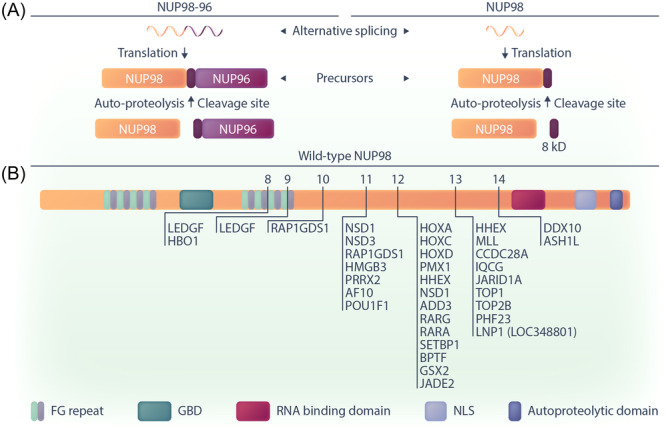
**Schematic representation of NUP98 expression and structure in cells**. **(A)** The expression involves alternative splicing and autoproteolytic cleavage, leading to the production of mature NUP98 and NUP96 proteins, as well as an 8 kD fragment. **(B)** The structure of the wild‐type NUP98 protein and position of NUP98 fusion breakpoints in leukemia. Arrows indicate exon numbers. GBD, Gle2‐binding domain; NLS, nuclear localization signal.

The NUP98 protein is a component of the nuclear pore complex (NPC), which is composed of over 30 different proteins. NPCs are transport channels that mediate the bidirectional transport of molecules (ions, polypeptides, mRNA, and proteins) between the nucleus and the cytoplasm by diffusion and active transport. *NUP98* and *NUP98‐NUP96* are two mRNA splice variants that are encoded by the *NUP98* gene. The NUP98‐NUP96 polypeptide undergoes cleavage, leading to the generation of a 90 kDa N‐terminal peptide and a 96 kDa C‐terminal peptide. Likewise, the precursor NUP98 polypeptide undergoes autoproteolytic cleavage, resulting in the generation of a 90 kDa N‐terminal peptide and an 8 kDa C‐terminal peptide (Figure 1).^21^, ^26^, ^27^

The presence of tandem phenylalanine‐glycine (FG) repeat domains is a hallmark of many NPC proteins.^28^ These intrinsically disordered regions (IDRs) form a mesh‐like structure and act as a selective barrier in the central channel of the NPC, preventing free exchange of molecules larger than 5 nm.^29^ In addition, by interacting with soluble nuclear transport receptors (NTRs) that bind FG repeats and cargo, these IDRs facilitate selective transport of molecules through the NPC. The N‐terminus of the NUP98 protein contains two FG/GLFG (Gly‐Leu‐Phe‐Gly) repeat regions with 38 repeats in total that are bisected by a Gle2‐binding sequence (GLEBS) domain.^30^ The FG repeats of NUP98 are essential for the maintenance of the NPC's entropic barrier and nucleocytoplasmic trafficking, primarily by interacting with NTRs such as CRM1 (chromosomal maintenance 1; also called Exportin 1 or Xpo1).^31^, ^32^, ^33^, ^34^, ^35^

While the NUP98 protein primarily resides in and interacts with components of the NPC including scaffold NUPs, fluorescence recovery after photobleaching (FRAP) experiments have shown that it is highly mobile and can also be detected in the nucleoplasm.^36^ The mobility of NUP98 mainly depends on its association with RNA polymerase II (RNAP II).^13^, ^14^, ^15^ The nucleoplasmic fraction of NUP98 is situated in self‐aggregated intranuclear clusters known as “GLFG” bodies and participates in gene expression or cell cycle regulation.^37^, ^38^, ^39^, ^40^, ^41^ The formation of GLFG bodies is linked to the involvement of FG repeats in the assembly of phase‐separated biomolecular condensates (explained further below), in which NUP98 proteins define their own protein interactome and engage in the regulation of gene expression regulation.^28^, ^42^, ^43^, ^44^ The involvement of NUP98 in transcriptional regulation occurs through its co‐localization with RNAP II, where NUP98 acts as a co‐transcriptional activator or repressor. This dual functionality arises from the association of the NUP98 FG repeats with various chromatin regulatory proteins.^23^, ^30^, ^45^, ^46^, ^47^, ^48^ The GLEBS domain of NUP98 is involved in RNA binding and transport through the nuclear envelope as well as cell cycle regulation and mitotic spindle formation mediated by the RNA export factor RAE1 (Gle2).^41^, ^49^ The C‐terminus of wild‐type NUP98 contains an RNA binding domain and a unique autoproteolytic cleavage site that is required for the production of the mature protein and is crucial for proper localization of the NUP98 protein in the NPC.^21^, ^49^ Using a C‐terminally truncated NUP98 variant that cannot bind to the NPC and is present in the nucleoplasm, Franks et al. showed that the Wdr82–Set1A/COMPASS (complex of proteins associated with Set1; WSC) is a binding partner for the NUP98 protein.^48^ WSC is a protein complex with roles in epigenetic regulation that promotes H3K4 trimethylation of specific genes.^50^ WSC is thought to be recruited by NUP98 to developmentally regulated genes to deposit H3K4me3 marks and induce gene expression.^48^, ^51^ In summary, the intricate balance maintained by NUP98 in cellular processes emphasizes its importance, and any shifts in this balance can have profound effects on normal cell physiology.

## *NUP98* FUSIONS ENCOMPASS MULTIPLE FUSION PARTNERS IN HEMATOLOGICAL MALIGNANCIES

The *NUP98* gene can be involved in genomic rearrangements with various partner loci.^18^ These fusions result from the junction of the 5′‐end of *NUP98* on chromosome 11p15 to the 3′‐end of the fusion gene partners.^18^ *NUP98::HOXA9* was the first described *NUP98* fusion in hematological malignancies.^52^, ^53^ NUP98::NSD1 is the most common NUP98 fusion protein, found in approximately 8% of pediatric AML patients.^54^ Along with NUP98::HOXA9 and NUP98::KDM5A (JARID1A) it is the most intensively studied NUP98 fusion protein. *NUP98*‐rearranged (*NUP98*‐r) AML is associated with poor prognosis, treatment failure, and high relapse rates.^8^, ^9^, ^14^, ^18^ The *NUP98* translocations can occur at any age but are more commonly observed in children, adolescents and young adults with a higher incidence in males with about 60% of cases.^8^, ^18^ Morphologically, *NUP98*‐r AMLs are linked to the M2/M4 FAB subtypes.^18^, ^55^ However, some of the *NUP98* fusions have a distinct phenotype and propensity to manifest in specific disease subsets. For example, AMLs harboring the *NUP98::KDM5A* fusion typically fall into the M6/7 category with no expression of the pluripotent markers CD34 and CD123.^18^ This particular fusion is linked to approximately 20% of childhood and adult cases with acute erythroid leukemia (AEL),^56^ and is also present in 10% of infant AMKL.^20^, ^57^ The *NUP98::NSD1* fusion is more common in childhood AML, accounting for 16% of normal karyotype pediatric leukemia^14^ and is predominantly found in M4/M5 AML subtypes.^18^, ^55^ When *NUP98* fusions occur alongside the *FLT3*‐ITD mutation, which happens in very high frequency in *NUP98::NSD1*, aberrant blasts often show signs of monocytic maturation, as evidenced by the expression of CD11b, CD36, and CD64.^18^ Certain *NUP98* fusions, such as those with *JADE2*, *RARA*, or *RARG* fusion partners, are associated with an acute promyelocytic leukemia phenotype (M3).^55^, ^58^, ^59^ Other *NUP98* fusions, while lacking a consistent immunophenotype, predominantly express markers characteristic of early progenitors.^18^ Notably, quantification of mRNA transcripts of the *NUP98* fusions can provide a targeted method for detecting molecular residual disease (MRD).^60^, ^61^

**Figure 2:**
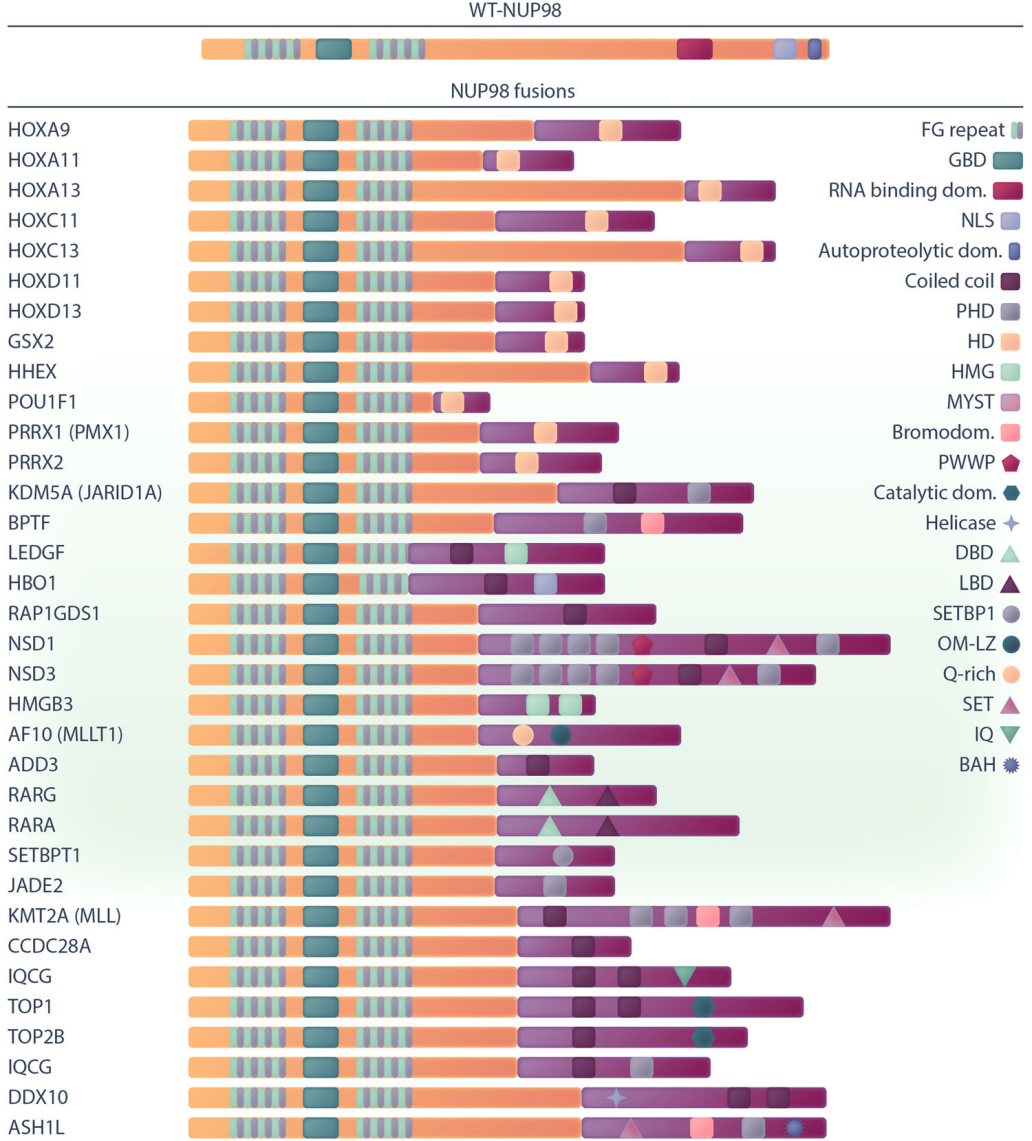
**Graphical representation of wild type NUP98 and its fusion partner structures, which involve functional domains for each fusion partner**. BAH, bromo‐adjacent homology; DBD, DNA binding domain; GBD, Gle2‐binding domain; HMG, high mobility group; LBD, ligand‐binding domain; NLS, nuclear localization signal; OM‐LZ, octapeptide motif‐leucine‐zipper; PWWP, Pro‐Trp‐Trp‐Pro; Q‐rich, glutamine‐rich; RNB, RNA‐binding domain. Functional domains for VRK1, LOC348801 (LNP1), FN1, and ANKRD28 have not been identified so far.

So far, more than 30 distinct fusion partners of *NUP98* have been reported (Figure 2 and Table 1). Considering the sub‐telomeric location of the *NUP98* breakpoint, traditional karyotyping may miss some of the *NUP98* fusions such as *NUP98::NSD1*, leading to their misclassification as AMLs with a cytogenetically normal karyotype.^14^, ^21^ Due to the rapid improvement of sequencing technologies more *NUP98* fusions have recently been revealed in monocytic, megakaryoblastic, and erythroid AML (French‐American‐British FAB M4, M5, M6, M7).^55^

**Table 1:** *NUP98* fusions in leukemia.

Fusion partner	Chromosome	Domain	Disease	Outcome	References
*HOXA9*	7p15	HD	AML, CML, MDS, CMML	Median OS: 13.5 months Median RFS: 6 months N fusion: 11 N total = 493	[53, 62, 63, 64, 65, 66]
*HOXA11*	7p15	HD	CML, JMML		[67, 68]
*HOXA13*	7p15	HD	AML, CML, MDS		[62, 67, 69]
*HOXC11*	12q13	HD	AML		[70]
*HOXC13*	12q13	HD	AML		[70, 71]
*HOXD11*	2q31	HD	AML		[72]
*HOXD13*	2q31	HD	AML, CML		[62, 73, 74]
*GSX2* (*Gsh2*)	4q12	HD	AML		[75]
*PRRX1* (*PMX1*)	1q23	HD	AML, CML, MDS		[23, 76, 77]
*PRRX2*	9q34	HD	AML		[78, 79]
*POU1F1*	3p11	HD	AML		[80]
*JADE2*	5q31	PHD	APL‐AML, MDS‐MPN, JMML		[81, 82]
*PHF23*	17p13	Coiled domain‐PHD	AML		[83]
*HHEX*	10q23	HD	AML		[84]
*NSD1*	5q35	SET‐PHD‐PWWP‐Coiled domain	MDS/MPN, AML, MPAL	CR: 38% OS: 36% EFS: 17% RR: 64% N fusion: 108 N total = 2235	[14, 18, 85, 86, 87, 88]
*NSD3*	8p11	SET‐PHD‐PWWP‐Coiled domain	MDS, AML		[24, 89]
*MLL* (*KMT2A*)	11q23	SET‐PHD‐Ciled domain‐SET binding domain‐Bromodoamin	AML‐MDS		[90]
*ASH1L*		SET, Bromo, PHD, BHD	AML, MDS		[91]
*SETBP1*	18q12	SET binding domain	T‐ALL		[92]
	17q23	PHD‐Bromodomain‐CC	AMKL‐ T‐ALL		[93, 94]
*KDM5A* (*JARID1A*)	12p13	PHD‐Jumonji (Demethylase)	AMKL	CR: 81% OS: 30% EFS: 25% RR: 68% N fusion = 32 N total = 2235	[18, 95]
*MLLT10* (*AF10*)	10p12	OM‐LZ, Q‐rich	MDS		[75]
ANKRD28*	3p25	Not identified	AML‐MDS		[96]
*DDX10*	11q22	Helicase domains‐CC	MDS, AML, CML		[97, 98]
*HMGB3*	Xq28	HMG box‐CC	AML		[99]
*KAT7* (*HBO1*)	17q21	MYST‐CC	CMML		[62, 100]
*PSIP1* (*LEDGF*)	9p22	HMG box‐CC	MDS, AML, CML		[21, 101, 102, 103, 104]
*RAP1GDS1*	4q21	CC	T‐ALL, AML		[105, 106]
*RARA*	17q21	DBD‐LBD	APL		[59]
*RARG*	12q13	DBD‐LBD	AML		[58]
*TOP1*	20q11	Topoisomerase‐Coiled domain	AML, MDS		[107, 108]
*TOP2B*	3p24	Topoisomerase‐ Coiled domain	AML		[109]
*VRK1*	14q32	Not identified	T‐ALL		[110]
*CCDC28A* (*C6orf80*)	6q24	Coiled domain	T‐ALL, AML		[111]
*IQCG*	3q29	Coiled domain, IQ	MPAL‐ T‐ALL		[25, 112]
*LOC348801* (LNP1)	3q12	Not identified	AML		[113]
*ADD3*	10q25	Coiled domain	T‐ALL, AML		[114]
*FN1*	2q31	Not identified	AML		[115]

Abbreviations: AML, acute myeloid leukaemia; CR, complete remission; MDS, myelodysplastic syndrome; N, number; OS, overall survival; RFS, relapse‐free survival; RR, relapse risk.

The fusion of NUP98 with other proteins often leads to a gain of function and the resulting fusion oncoproteins inherits parts of the properties and functionalities of both NUP98 and its partner. Although functional domains have not been identified in all fusion partners, many of them encode homeodomain DNA‐binding motifs (HD), including clustered (*HOXA9*/*11*/*13*, *HOXC11*/*13*, *HOXD11*/*13*) and nonclustered (*HHX*, *GSX2*, *PRRX1*, *PRRX2*, *POU1F*) *HOX* genes. Additionally, there are various non‐HOX fusion protein partners for NUP98, which possess DNA‐ or chromatin‐binding domains or domains with other functions (detailed in Table 1). Notably, certain fusion partners such as NSD1, NSD3, and KMT2A (MLL) harbor multiple functional domains. These encompass both the chromatin‐binding plant homeodomain (PHD), enabling H3K4me3 binding, and the catalytic lysine methyltransferase SET (Su(var)3‐9, Enhancer‐of‐zeste, and Trithorax) domain, mediating histone methylation. Additionally, *NUP98* fusions frequently co‐occur with a set of other recurrent somatic mutations, the most prevalent being *FLT3*‐ITD and *WT1*. For example, 74% of patients with *NUP98::NSD1* harbor the *FLT3*‐ITD mutation, and 42% possess the *WT1* mutation.^18^ These additional mutations can confer proliferative advantages to the leukemic cells and contribute to leukemogenesis (Table 2).

**Table 2:** Common co‐occurrent mutations with *NUP98* fusions in AML.

Co‐occurrent mutations	Fusion partner	References
*FLT3*‐ITD	*NSD1*, *KDM5A*, *LNP1*, *HOXC11*, *HOXA9*, *HOXA13*, *HOXD13*, *NSD3*	[14, 18, 116, 117, 118]
*WT1*	*NSD1*, *KDM5A*, *TOP1*, *HOXC11*, *HOXA9*, *HOXD11*, *NSD3*, *HOXA13*, *HOXA11*	[14, 18, 116, 118]
*NRAS*	*NSD1*, *HOXA9*, *HOXA11*, *DDX10*	[14, 56, 118]
*KRAS*	*HOXA9*, *HOXD11*	[11, 118]
*RB1*	*KDM5A*	[62, 119]
*NOTCH1*	*RAP1GDS1*	[117]
*MYC*	*NSD1*	[62, 120, 121]
*KIT*	*HOXC11*, *HOXA9*, *NSD3*, *NSD1*	[118]
*RUNX1*	*NSD1*, *RARG*	[122, 123]
*ASXL1*	*NSD1*	[124]
*MYB*	*RAP1GDS1*	[117]

## NUP98 FUSION PROTEINS FORM ABERRANT BIOMOLECULAR CONDENSATES

**Figure 3:**
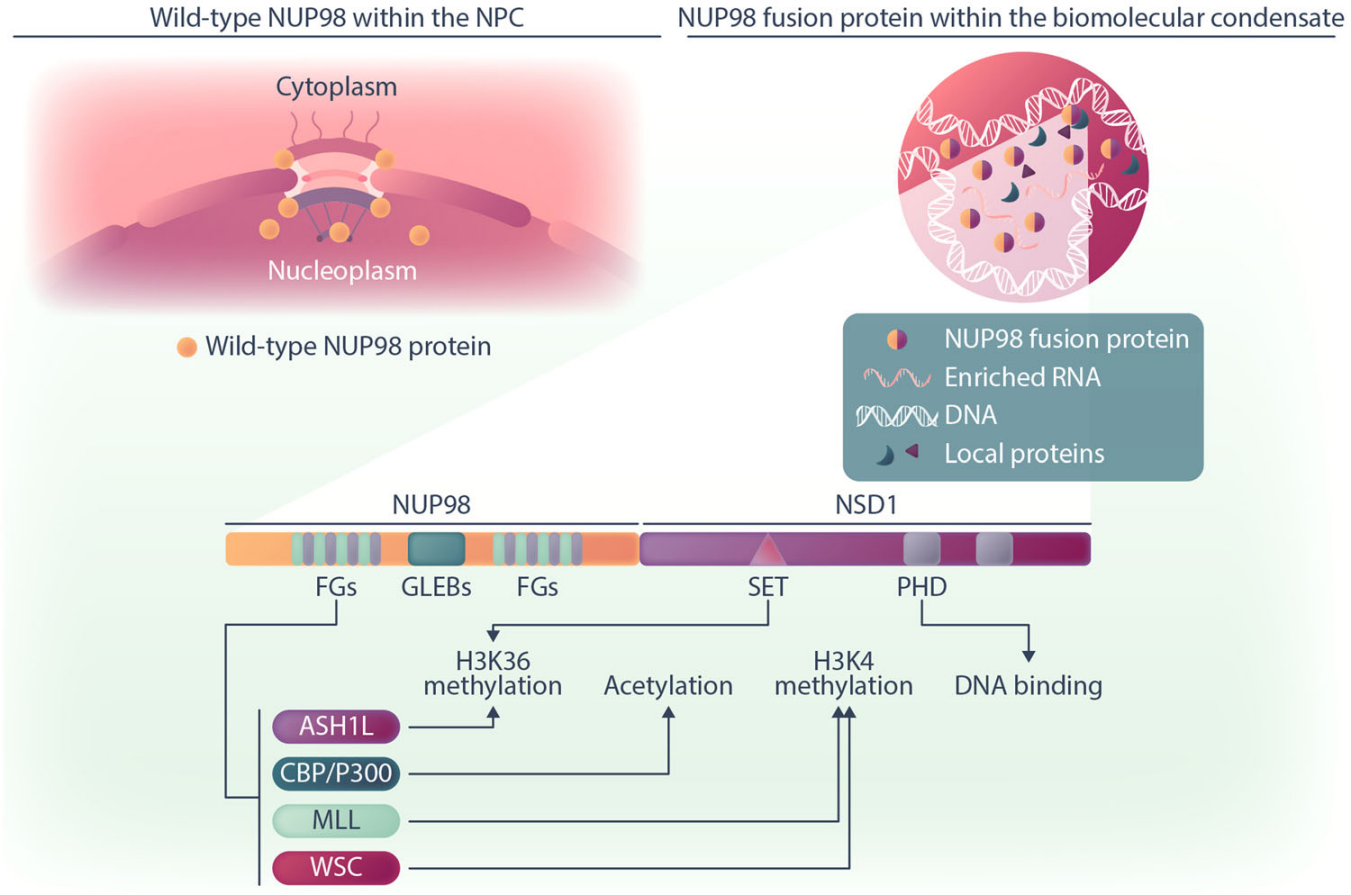
**Schematic illustration of the NUP98 protein in healthy cells and interactions of NUP98 fusion proteins in AML cells**. The mature wild‐type NUP98 protein is predominantly located in both the nucleus and cytoplasmic regions of the NPC, as well as in the nucleoplasm. The NUP98 fusion proteins are predominantly observed within nuclear punctate structures, known as biomolecular condensates, distributed throughout the nucleoplasm. Within these condensates, NUP98 fusions form their distinct protein interactome, where the N‐terminus FG repeats engage with various regulatory proteins. Specifically, the FG repeats interact with MLL, WSC, CBP/P300, and ASH1L protein complexes, all of which play roles in epigenetic regulation. Simultaneously, functional domains such as PHD and SET in fusion partners like NSD1 collaborate with this regulatory function or engage with chromatin. Figure Made in BioRender.com.

Despite intensive research, the molecular mechanisms underlying NUP98 fusion‐induced oncogenesis have remained incompletely understood. Recent results indicate that NUP98 fusion proteins show a different subcellular distribution than wild‐type NUP98 and form aberrant biomolecular condensates that affect chromatin architecture and gene expression, ultimately leading to AML development.^125^, ^126^ Various studies have demonstrated that NUP98 fusion proteins localize to nuclear puncta and promote the formation of aberrant biomolecular condensates.^125^, ^127^, ^128^, ^129^ This altered cellular distribution has been attributed to the lack of a C‐terminally located NPC‐targeting signal in the fusion protein (Figure 3).^30^ Fluorescence recovery after photobleaching (FRAP) experiments performed with NUP98::HOXA9 and NUP98::NSD1 demonstrated the liquid‐like nature of the phase‐separated droplets formed by these two fusion proteins.^128^, ^129^

Given the important roles of NUP98 fusion proteins in the context of biomolecular condensates, the investigation of the fusion protein interactome has provided exciting new insights into the molecular mechanisms of leukemogenesis driven by NUP98 fusion proteins. Affinity purification coupled to mass spectrometry (AP‐MS) experiments revealed a minimal overlap between the interactors of NUP98 fusions and those of the wild‐type NUP98 protein.^125^ While NUP98 was interacting with components of the NPC and proteins involved in nucleocytoplasmic transport, NUP98 fusion proteins were predominately interacting with RNA‐binding proteins and RNA helicases. In line with this, the core interactome of distinct NUP98 fusion proteins featured factors with roles in RNA splicing, ribosome biogenesis and transcriptional control. In addition, the core interactome of NUP98 fusion proteins was highly enriched for biomolecular condensation‐related proteins, including FUS, HNRNPA1 and GAR1.^125^ Terlecki‐Zaniewicz et al. showed that expression of NUP98 fusion proteins significantly changed the composition of biomolecular condensates. Proteins that were recruited into phase‐separated structures in a NUP98 fusion‐dependent fashion were enriched for complexes involved in transcriptional activation and chromatin organization.^125^

Notably, the nature of NUP98 fusion protein‐containing biomolecular condensates and their core interactome are dependent on the FG repeats in the N‐terminal part of NUP98. Deletion of FG repeats in NUP98::HOXA9 abrogated puncta formation and the ability of leukemic transformation of mouse hematopoietic stem and progenitor cells (HSPC) in vitro and in vivo, emphasizing the essentiality of the FG repeat regions for oncogenesis.^127^, ^128^ Remarkably, replacing the NUP98 N‐terminus by an artificial stretch of 39 FG repeats fused to the C‐terminus of KDM5A (artFG‐KDM5A) preserved its localization to nuclear puncta and maintained its oncogenic function by inducing leukemia‐associated gene expression programs similar to the original NUP98::KDM5A fusion protein.^125^

Within the setting of biomolecular condensates induced by NUP98 fusions, it has been shown that mutation of the FG motifs in the NUP98 N‐terminus led to the loss of the interaction between NUP98::NSD1 and the SMARCA5 protein, a core component of the Nucleosome Remodeling Factor (NURF) complex.^129^ Furthermore, the interaction of SMARCA5 with NUP98::NSD1 was shown to be crucial for NUP98::NSD1‐driven leukemic transformation. These data highlight the importance of IDR‐mediated phase separation for the maintenance of leukemogenic transcriptional programs by NUP98 fusion proteins.

The fusion partners of NUP98 also affect condensate formation and composition. Mutation of the DNA‐binding homeodomain of HOXA9 in the context of the NUP98::HOXA9 fusion oncoprotein resulted in fewer but larger condensates that showed less overlap with DNA. This indicates that not only homotypic interactions between the intrinsically disordered regions (IDRs) of the NUP98 N‐terminus are important for proper formation of oncogenic condensates, but also heterotypic interactions between the HOXA9 DNA‐binding domain and chromatin.^127^, ^128^ However, the PHD chromatin‐binding domains of NUP98::NSD1 might play a less important role, as their deletion did not affect puncta formation or global gene expression patterns.^129^

The formation of aberrant condensates has functional consequences for chromatin organization. Ahn and colleagues investigated the effect of NUP98‐IDR‐mediated phase separation on NUP98::HOXA9 chromatin occupancy and global chromatin architecture.^127^ Chromatin immunoprecipitation followed by high‐throughput sequencing (ChIP‐seq) showed that mutations in the NUP98 IDR impaired chromatin binding of NUP98::HOXA9. While the non‐mutated NUP98::HOXA9 fusion protein bound to genes associated with developmental processes and leukemia, inducing *HOX* genes, *PBX3* and *MEIS1* in the context of regions decorated with the activating H3K27ac histone mark, this pattern was lost when the NUP98 N‐terminal IDR was mutated. Replacing the NUP98 N‐terminus with the phase‐separation‐prone IDR of the FUS protein induced chromatin binding patterns similar to those of NUP98::HOXA9. Furthermore, analysis of changes in the three‐dimensional chromatin structure revealed 232 specific chromatin loops that were dependent on the intact FG repeat regions of the NUP98::HOXA9 fusion protein. Interestingly, the respective loop anchors overlapped with NUP98::HOXA9 binding sites but not with CTCF binding sites. These observations support a phase separation‐driven mechanism of NUP98‐fusion chromatin binding and chromatin looping that is independent of CTCF.^127^ Altogether, these studies suggest that the conserved N‐terminus of NUP98 fusion oncoproteins plays a pivotal role in the initiation and maintenance of leukemogenesis via the formation of phase separation‐mediated biomolecular condensates. However, as several studies used ectopic expression of fusion genes at potentially nonphysiological levels and/or nonhematopoietic cell models, further validation of this concept is warranted.

Several studies have shown that interactions between NUP98 fusion proteins and other chromatin binders result in the recruitment of NUP98 fusion proteins to defined genomic regions such as the *HOXA* locus. Although not explored in the context of biomolecular condensates, these interactions are highly likely to occur within condensed cellular structures. For instance, the FG repeats of the NUP98::HOXA9 fusion protein interact with chromatin pre‐bound CRM1, resulting in selective recruitment of the fusion protein to the *HOXA* cluster region and subsequent activation of gene expression. This induction of *HOX* gene expression has been attributed to the ability of the NUP98::HOXA9‐CRM1 complex to change chromatin structure.^130^ Moreover, the FG repeats of multiple NUP98 fusion proteins can interact with the KMT2A protein complex, and this interaction is essential for the oncogenic activity of the fusion proteins (Figure 3).^30^, ^46^, ^131^ The KMT2A protein complex recruits NUP98 fusion proteins to its target loci and induces trimethylation of H3K4 through the C‐terminal SET domain of KMT2A, thereby converting promoters into an active state.^30^ These findings are consistent with previous work showing the association of NUP98‐FG repeats with the CBP/p300 protein complex on active genes.^47^ In line with distinct intracellular distribution, wild‐type NUP98 cannot interact with KMT2A. Given that both KMT2A and NUP98 fusion oncoproteins induce similar gene signatures in AML and that the KMT2A protein plays a role in recruiting NUP98 fusion proteins to particular gene loci, more investigation is required to determine whether and to what extent the identity of NUP98 fusion protein target genes is defined by the KMT2A protein complex or NUP98 fusions. In summary, NUP98 fusion proteins establish a complex network of protein interactions, likely within biomolecular condensates, which contributes to the specific transcriptional output that drives leukemic transformation.

## SHAPING TRANSCRIPTIONAL PROGRAMS BY NUP98 FUSION PROTEINS

Phase separation can regulate transcription by concentrating IDR‐containing transcription factors and coactivators and inducing proximity between super‐enhancers and promoters.^132^, ^133^ These and similar observations contributed to the emergence of models that link phase separation to transcriptional factories. This implies that transcription occurs in non‐random locations throughout the nucleus and might be controlled by the local concentration of the transcriptional machinery in subnuclear compartments.^133^, ^134^ Numerous studies have consistently associated *NUP98*‐r AML with the overexpression of a specific subset of pivotal genes with important functions in leukemia. This set includes *HOXA/B*, *MEIS1*, *MEF2C*, *PBX3*, *CDK6*, *FLT3* and *IGF2BP2*, all of which play critical roles in the regulation of proliferation and differentiation processes in hematopoietic cells.^18^, ^54^, ^116^, ^135^, ^136^, ^137^ There is also a notable overlap in the transcriptional program of *NUP98*‐r AMLs with those found in *NPM1*‐mut, *KMT2A*‐r, and *UBTF‐TD* AMLs. This overlap contributes to the co‐clustering of *NUP98*‐r AMLs with these specific AML subtypes, suggesting shared pathways and disease mechanisms.^116^, ^136^, ^138^, ^139^, ^140^ In line with the model of condensation‐dependent transcriptional regulation, mutation of the N‐terminal IDR in NUP98 fusion oncoproteins has significant effects on global gene expression. While the expression of NUP98::HOXA9 caused differential expression of almost 900 genes, including *HOXA* genes, *PBX1* and *MEIS1*, expression of the IDR‐mutated NUP98::HOXA9 only induced the deregulation of 61 genes.^127^

Moreover, based on their global gene expression, AMLs with *NUP98* fusions cluster in two groups, comprising either AMKLs or myelo‐monocytic AML types.^140^ As described before, interaction with proteins such as CRM1 and KMT2A direct NUP98 fusion proteins to their target gene loci, including the *HOXA* gene cluster, where their association with chromatin is enhanced through the functional domains in the C‐terminus of the fusion protein.^30^, ^141^ Many NUP98 fusion partners possess DNA or chromatin binding domains such as HD (homeodomain), PHD (plant homeodomain), or PWWP (Pro‐Trp‐Trp‐Pro). For example, NUP98 fusions such as NUP98::NSD1, NUP98::KDM5A and NUP98::PHF23 have a PHD domain at the C‐terminus which can recognize and bind to H3K4me2/3, a mark of open active chromatin.^21^, ^142^ In this context, deletion or mutation of the PHD domain inhibits H3K4me binding activity and reduces the expression of critical transcriptional factor genes such as *HOX* genes, *GATA3, MEIS1, EYA1*, and *PBX1*. This, in turn, leads to the inhibition of leukemogenesis in an AML mouse model.^142^ The H3K4me3 binding ability of PHD domains may serve as a seed for recruiting other proteins with roles in epigenetic regulation to induce spreading of the H3K4me3 mark on chromatin. Simultaneously, regulatory domains within the fusion protein partners can also actively participate in gene activation. For example, the catalytic SET domain of the NSD1 protein in the NUP98::NSD1 fusion is involved in the mono‐ and dimethylation of H3K36 in intergenic regions, a feature of actively transcribed genes such as the *HOXA* gene cluster.^143^, ^144^ Similarly, the DNA binding activity of the HD fusion partners of NUP98 such as HOXA9 has been highlighted as a crucial factor influencing aberrant gene expression.^145^, ^146^

The transcriptional programs downstream of NUP98 fusion proteins are induced and/or re‐enforced by transcription factors with important functions in hematopoiesis and leukemia. Recent studies in this context underscore the crucial role of MEIS1 in *NUP98*‐r AML. *MEIS1* is an early target gene of *NUP98* fusion expression.^116^, ^136^ Together with HOXA9 and PBX3, MEIS1 forms a trimeric protein complex that is highly expressed in immature hematopoietic stem cells. Overexpression of *MEIS1/PBX3* or *MEIS1/HOXA9* is sufficient for leukemic transformation of mouse hematopoietic stem cells and is linked to the development of various AMLs with poor prognosis.^139^, ^147^, ^148^, ^149^, ^150^ An important function of MEIS1 is to regulate expression of *FLT3*, another gene that is highly expressed in *NUP98*‐r AMLs.^137^, ^151^ Signaling via the FLT3 receptor is a crucial molecular pathway that actively supports survival and stimulates cell proliferation of hematopoietic stem and progenitor cells. Previous findings indicate that AML patients with *FLT3*‐ITD had significantly different outcomes based on co‐occurring mutations.^152^ Individuals with *FLT3*‐ITD and favorable risk mutations in *NPM1*, *CEBPA*, t(8;21), or inv(16) had a superior 5‐year Event‐Free Survival (EFS) of 64% compared to those of 22% for patients harboring poor‐risk mutations such as *WT1*, *UBTF*, or *NUP98::NSD1*.^152^ The prevalence of the *FLT3*‐ITD mutation in *NUP98*‐r AML is associated with poor prognosis and induction failure.^54^, ^137^ This observation hints at a potential collaboration and interplay between MEIS1 and FLT3 in the context of *NUP98* fusion‐driven leukemia.

In summary, differences in the transcriptional programs in cells harboring NUP98 fusions versus cells expressing wild‐type NUP98 can be attributed to aberrant distribution of the fusion oncoprotein within the nucleus and the presence of a chromatin binding and/or ‐regulatory moiety such as PHD, HD, and SET domains in the C‐terminus of the fusion partners. These domains mediate a strong and stable association of the NUP98 fusion protein with chromatin, which is absent in the wild‐type NUP98 protein. Strong association with chromatin increases the recruitment of other regulatory protein partners of NUP98 fusions, leading to activation or repression of target genes.^23^, ^48^, ^146^, ^153^ However, the mechanism through which different NUP98 fusions, involving partners possessing chromatin binding domains like NSD1 or lacking such domains like DDX10, execute a similar transcriptional program has remained unclear.^97^, ^144^

## THERAPEUTIC OPPORTUNITIES FOR NUP98‐r AML

Despite significant progress in understanding the molecular mechanism of NUP98 fusions and their concomitant genetic alterations in oncogenesis, finding effective therapeutic strategies for this AML subtype is still a significant challenge. Genomic heterogeneity and somatically acquired mutations are major factors that may impact treatment outcome. The presence of *NUP98* fusions is associated with an adverse clinical outcome. Within a pediatric AML cohort, it has been observed that 72% of AML patients with *NUP98* fusions were refractory to induction therapy.^8^ Notably, the outcomes for *NUP98*‐r AML patients can vary depending on the type of fusion partner involved. A study of 2235 children and young adults showed that while the overall CR rate for *NUP98*‐r AML patients was 50% after initial induction therapy, patients with *NUP98::NSD1* fusions had a substantially lower CR rate of 38%. This was in contrast to an 80% CR rate for patients with *NUP98::KDM5A* fusions.^18^ After achieving CR in the first or second course of induction therapy, these AML patients often undergo HSCT to reduce the risk of relapse. However, various studies have reported that more than 25% (25%–70%) of *NUP98*‐r AML patients experience relapse even after HSCT.^8^, ^95^, ^154^, ^155^ Notably, it has been observed that patients with *NUP98::HOXA9* fusions have lower relapse rates when receiving HSCT after their first CR (25%) compared to those undergoing transplantation after their second CR (45%).^154^ Considering the toxicity of chemotherapy and the high rate of relapse in this AML subgroup, novel targeted approaches may provide interesting options to improve patient outcomes. Insights into the composition of NUP98 fusion protein complexes, the interaction with secondary mutations such as *FLT3*‐ITD and the fusion oncoprotein‐induced transcriptional networks have identified multiple candidates for a targeted intervention.

### TARGETING NUP98 FUSION PARTNER MOIETIES

**Figure 4:**
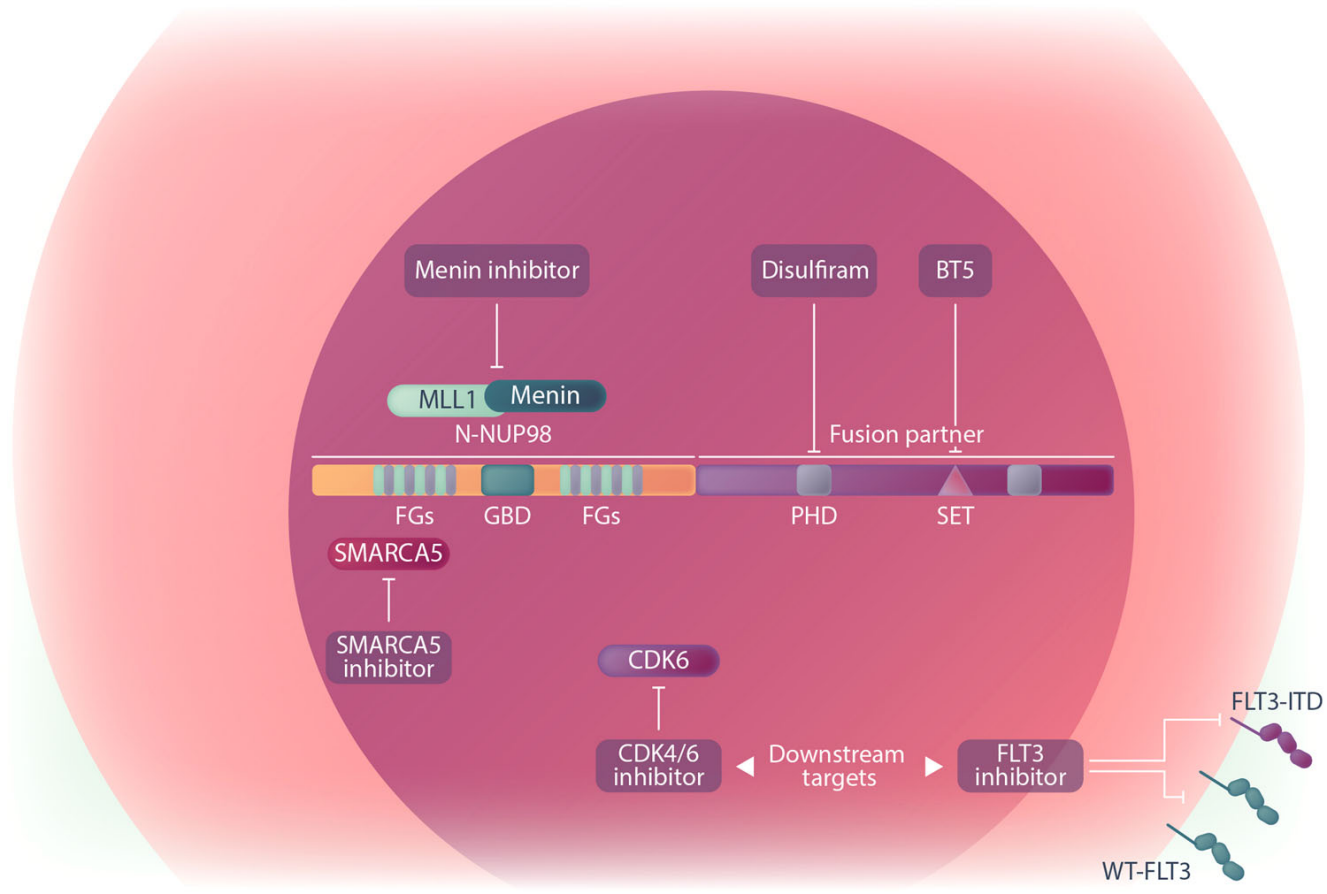
Graphical depiction of treatment opportunities for NUP98‐r AML.

Several studies have shown that the fusion protein partners of NUP98 play important roles in leukemogenesis.^21^, ^142^, ^144^ To this end, researchers have attempted to block the activity of the functional domains in the NUP98 fusion partner sequences. One of the challenges is that most transcriptional regulators which serve a critical role in driving leukemia are difficult to drug due to a lack of deep protein pockets or large protein–protein interaction (PPI) interfaces.^156^ However, covalent inhibition of the NSD1 histone methyltransferase has been shown to exert an antileukemic effect against *NUP98::NSD1* leukemia cells.^144^ The NSD1 inhibitor BT5 blocked the histone methyltransferase activity of the NSD1 SET domain and suppressed H3K36 dimethylation in *HOXA* genes, subsequently impairing the expression of target genes, cell proliferation, and colony‐forming activity.^144^ Another strategy is to target the PHD domain, which is located in the C‐terminus of different NUP98 fusion oncoproteins. Blocking the PHD domain of NUP98::PHF23 and NUP98::KDM5A fusion proteins by the FDA‐approved drug disulfiram has been shown to disrupt the H3K4me3 binding potential of fusion proteins, impair expression of *HOXA/B*, *MEIS1* genes and increase cell death in AML mouse cells (Figure 4).^83^ It has been previously shown that disulfiram also impairs leukemia progression in *KMT2A*‐r AML by inhibiting the CXXC domain (Cysteine, X, X, Cysteine), a crucial DNA‐binding motif found in KMT2A fusion proteins.^157^ Consequently, Disulfiram might be useful for the treatment of a wider group of AML subtypes driven by oncoproteins that possess chromatin or DNA binding domains.

### TARGETING NUP98 FUSION PROTEIN INTERACTIONS

Another strategy for the treatment of *NUP98*‐r AML is the targeting of NUP98 fusion protein interactions. In *NUP98*‐r, *KMT2A*‐r, and *NPM1*‐mutant leukemia, all of which induce and depend on the *MEIS1/HOXA* transcriptional program, small molecule inhibitors that disrupt the Menin‐KMT2A interaction have demonstrated potent antileukemic effects.^116^, ^136^, ^158^ Generally, the KMT2A cofactor Menin plays a crucial role in recruiting KMT2A to the promoter regions of key target genes, such as *MEIS1*. Similarly, NUP98 fusion proteins also interact with the KMT2A protein complex at its target genes, including *MEIS1* and *HOXA9*. In *NPM1*‐mutant AML, the Menin‐KMT2A interaction is also vital in shaping the leukemic gene signature.^136^, ^158^, ^159^, ^160^ Additionally, *UBTF*‐TD AML demonstrates transcriptional profiles similar to those observed in *NUP98*‐r, *KMT2A*‐r, and *NPM1*‐mutant AMLs, marked by a significant overlap of overexpressed genes.^138^, ^140^, ^161^, ^162^ Thus, these AML (*KMT2A*‐r, *NUP98*‐r, *UBTF*‐TD, and *NPM1*‐mutant) subtypes share a similar transcriptional program with Menin playing the role of a conductor. Sensitivity of *KMT2A*‐r and *NPM1*‐mut AMLs to Menin inhibition has been documented previously.^158^, ^159^, ^160^, ^163^, ^164^ Notably, this sensitivity is abolished by mutations in the *MEN1* gene, further confirming the critical interaction between Menin and KMT2A.^165^ In a study, we recently demonstrated that AMLs harboring *UBTF*‐TD are also sensitive to Menin inhibition, and that this manifests in similar gene expression changes.^162^ Heikamp et al. showed that *NUP98*‐r AML cells are Menin‐dependent and disrupting the Menin‐KMT2A interaction with the Menin inhibitor VTP50469 induced anti‐proliferative effects and survival benefits in mouse AML models driven by *NUP98::NSD1* and *NUP98::KDM5A*.^136^ By using in vivo and ex vivo models, we also showed that Menin inhibition via revumenib is effective in halting leukemic progression of *NUP98::NSD1* (*FLT3*‐ITD+) and *NUP98::TOP1* (*WT1*+) AMLs, regardless of concomitant mutations. Furthermore, combined Menin and FLT3 inhibition exerted a synergistic effect in suppressing *NUP98::NSD1* expressing AML cells.^116^ However, the effectiveness of Menin inhibition in *NUP98*‐r AMLs has only been studied in a limited number of NUP98 fusions. Further investigation is necessary to fully assess the potential therapeutic impact of Menin inhibitors across a broad spectrum of NUP98 fusion variants and AML subtypes. The promising preclinical results with Menin inhibitors have led to the initiation of first‐in‐human clinical trials for revumenib, ziftomenib, and JNJ‐75276617 in adults with relapsed/refractory (R/R) leukemia featuring *KMT2A*‐r and *NPM*−1 mutations. Subsequently, *NUP98*‐r AMLs have also been included in some of these clinical trials (Table 3). The early result of phase I/II study investigating oral combination of revumenib with decitabine/cedazuridine (ASTX727) and venetoclax has demonstrated high efficacy in AML patients with KMT2A‐r, NPM1 mutations, and NUP98‐r.^166^ A phase I trial is currently underway to assess the safety and tolerability of the Menin inhibitor JNJ‐75276617 (developed by Janssen Pharmaceuticals) as both monotherapy and in combination with chemotherapy (NCT05521087) for AML patients with *KMT2A*‐r, *NUP98*‐r, and *NPM1* mutations. Ziftomenib (KO‐539) is also under evaluation as monotherapy or in combination with chemotherapy for assessing its efficacy in *KMT2A*‐r and *NPM1*‐mutant AML. Notably, however, Menin inhibitors in clinical use may give rise to toxicities, as exemplified by the differentiation syndrome observed in trials all Menin inhibitors when used as monotherapy.^167^

**Table 3:** Menin inhibitors in clinical trials.

Compound	Trial	Combination	Eligibility	Participant	Status	Phase
Revumenib (Syndax Pharmaceuticals)	NCT04065399 (Augment‐101)	Monotherapy	R/R *KMT2A*‐r AML/ALL/MPAL, *NPM1c*, *NUP98*‐r AML	Child, Adult, Older Adult	Recruiting	I–II
	NCT05326516 (AUGMENT‐102)	Combination with FLA chemotherapy	R/R *KMT2A*‐r, *NPM1c*, *NUP98*‐r AML/ALL/MPAL	Child, Adults	Not yet recruiting	I
	NCT06226571	Chemotherapy, HiDAC	*KMT2A*‐r, *NPM1c*, *NUP98*‐r AML	Adult, Older Adult	Not yet recruiting	I
	NCT06222580	Gilteritinib	R/R *FLT3* mutated AML with *KMT2A*‐r, *NUP98*‐r and *NPM1c* alterations	Adult, Older Adult	Not yet recruiting	I
	NCT05360160	Decitabine/cedazuridine (ASTX727) and venetoclax	R/R MPAL/AML with *KMT2Ar*, or *NUP98r*, or *NPM1c*	Child, Adult, Older Adult	Recruiting	I–II
	NCT06229912	Monotherapy	Leukemia Associated With Upregulation of *HOX* genes, including *NUP98*‐r AML	Child, Adult, Older Adult	Not yet recruiting	II
	NCT06177067	Revumenib, azacitidine, and venetoclax	R/R *KMT2A*‐r, *NPM1c*, *NUP98*‐r, *PICALM*::*MLLT10*, *DEK*::*NUP214*, *UBTF*‐TD, *KAT6A*::*CREBBP*, *SET*::*NUP214* AML	Child, Adult	Not yet recruiting	I
Ziftomenib (Kura Oncology)	NCT06376162	FLA chemotherapy	R/R *KMT2A*‐r/*NUP98*‐r/*NPMc* Acute leukemia	Child 0–21 years	Not yet recruiting	I
JNJ‐75 276 617 (Janssen)	NCT05521087	AML: JNJ‐75 276 617/FLA ALL: JNJ‐75 276 617/DEX/VCR/PEG	R/R *KMT2A*‐r, *NPM1c*, *NUP98* or *NUP214* alterations Acute leukemia	Child, Adult	Not yet recruiting	I
	NCT04811560	Monotherapy	R/R *KMT2A*‐r, *NPM1c*, *NUP98* or *NUP214* alterations Acute leukemia	Adult, Older Adult	Recruiting	I

*Note*: http://clinicaltrials.gov/

Abbreviations: chemotherapy, Cytarabine and Daunorubicin; DEX, Dexamethasone; FLA, Fludarabine, Cytarabine; HiDAC, high‐dose cytarabine; MPAL, mixed‐phenotype acute leukemia; PEG, PEG‐Asparaginase; VCR, Vincristine.

In this context, AML subtypes that display a transcriptional similarity and co‐cluster with *KMT2A*‐r, *NPM1*‐mutant, and *NUP98*‐r AMLs, including those harboring *UBTF*‐TD and *DEK::NUP214* may also show increased sensitivity towards Menin inhibition.^140^ This hypothesis is further supported by our recent finding that *UBTF*‐TD AML is highly sensitive to Menin inhibition.^162^

While the exact mechanisms by which NUP98 fusions regulate protein interactions in the nucleoplasm or biomolecular condensates are not fully understood, disrupting the formation of nuclear foci or the functional interactions of associated proteins presents another potential avenue for targeting leukemia cells. In *NUP98::NSD1* AML cells, targeting the transcriptional co‐regulator SMARCA5, which has been reported as a critical partner of NUP98 fusions in biomolecular condensates, has already been addressed.^129^ As describe previously, CRM1 is involved in recruiting NUP98 fusions to its target sites and providing access chromatin for *HOX* gene regulation. CRM1 inhibitors such as leptomycin B and selinexor, which has obtained FDA approval for the treatment of adult patients with multiple myeloma, could potentially be effective in *NUP98*‐r AML.^62^, ^129^, ^168^

### TARGETING NUP98 FUSION PROTEIN DOWNSTREAM FACTORS AND CO‐OPERATIVE MUTATIONS

Another potential treatment strategies for *NUP98*‐r is targeting factors that are induced by NUP98 fusion proteins, as the majority of NUP98 fusions exploits similar downstream targets to fuel leukemia.

Among NUP98 target genes, loss of *CDK6* has a significant impact on NUP98 fusion‐driven leukemogenesis, a feature shared with other AML subtypes such as *KMT2A*‐rearranged or *RUNX1::RUNX1T1*‐positive AMLs.^135^, ^169^, ^170^ The CDK4/6 inhibitor palbociclib, which is approved for the treatment of breast cancer, demonstrated potent anti‐proliferative activity and differentiation induction in murine AML cells driven by *NUP98::KDM5A*, *NUP98::DDX10*, and *NUP98::NSD1*, as well as in human *NUP98*‐r leukemic cells.^135^ Furthermore, CDK6 is reported as a critical factor for the survival of AML cells with *FLT3*‐ITD mutations, where FLT3‐ITD signaling is the primary cause of CDK6 overexpression through a pathway involving the SRC‐family kinase HCK.^171^ As *FLT3*‐ITD is a common recurrent mutation in *NUP98*‐r AML, targeting CDK6 may be a promising therapy option for this AML subtype.

*FLT3*‐ITD itself is a clinically highly relevant mutation that frequently co‐occurs with *NUP98* rearrangements. Currently, different FLT3 inhibitors are used for the treatment of patients with FLT3 mutations, including midostaurin, sorafenib, quizartinib, crenolanib, and gilteritinib. Based on type and generation, FLT3 inhibitors have different specificities and target active or inactive states of the FLT3 protein in cells with *FLT3*‐ITD or *FLT3* kinase domain point mutations (*FLT3*‐TKD). A type 1 inhibitor such as gilteritinib inhibits both the TKD and the ITD mutations, whereas a type 2 inhibitor such as quizartinib solely targets the ITD mutation, but not the TKD.^172^, ^173^ A study using mouse bone marrow progenitor cells co‐expressing *NUP98::NSD1* and *FLT3*‐ITD reported that these cells are more sensitive to midostaurin compared to cells expressing either aberration alone, highlighting the defining role of the FLT3 signaling pathway in *NUP98::NSD1*‐driven AML.^174^ However, during monotherapy with FLT3 inhibitors loss of the *FLT3*‐ITD mutation has been reported in relapsed patients, indicating the selective outgrowth of minor clones without *FLT3* mutations under selective pressure.^175^ Furthermore, after treatment with FLT3 inhibitors, de novo resistance mutations may arise in the FLT3 molecule.^175^, ^176^, ^177^ This process may result in the development of resistance followed by relapse. Thus, the best strategy may be to use inhibitors with a more narrow resistance profile (e.g., gilteritinib) or applying combination therapy with chemotherapeutic agents, HSCT, MEK inhibitors, hypo‐methylating agents (HMA), or CDK6 inhibitors to maximize the survival benefit for AML patients.^171^, ^178^, ^179^, ^180^, ^181^, ^182^, ^183^, ^184^

Combination therapies that include BCL2 inhibitors, such as venetoclax, paired with cytotoxic or hypomethylating agents, present a promising treatment strategy for *NUP98*‐r AML, despite the limited clinical data available for this subgroup. Venetoclax has shown significant efficacy in AMLs with *KMT2A*‐r and *NPM1* mutation.^185^, ^186^, ^187^ This effectiveness is partly attributed to the link between BCL‐2 inhibitor efficacy and the overexpression of *HOXA/B* genes.^188^ Although applied in a limited number of cases, it has been observed that patients with *NUP98::NSD1* (and *FLT3‐ITD*+) who respond poorly to chemotherapy may benefit from the inclusion of venetoclax and FLT3 inhibitors in their treatment regimens.^189^, ^190^, ^191^ The transcriptional similarities between *KMT2A*‐r and *NPM1*‐mutant AMLs and *NUP98*‐r AMLs suggest that venetoclax combinations might also be effective in treating AMLs with *NUP98* fusions, particularly those with high *BCL2* expression such as *NUP98::NSD1*, which are typically resistant to conventional chemotherapy. Currently, the efficacy of combining the Menin inhibitor revumenib, venetoclax, and a hypomethylating agent is being investigated in children with relapsed/refractory AML in a phase I/II, investigator‐initiated trial (NCT05360160).^166^ Furthermore, It has been demonstrated that the combination of the BCL2‐inhibitor navitoclax and the SRC/ABL‐inhibitor dasatinib has synergistic effects on patients and engineered cell models with *NUP98::NSD1* and *FLT3*‐ITD.^183^ The authors of this study speculate that enhanced expression of *LCK*, *FGR*, and *BCL2A1* in *NUP98::NSD1*+, *FLT3*‐ITD+ patients may be the cause of this synergistic effect. In addition, there is a report of a patient with the *NUP98::NSD1* fusion, along with concomitant *IDH1* and *GATA2* mutations, who did not achieve CR despite two courses of chemotherapy, but the combination of venetoclax and decitabine led to complete remission.^191^ Taken together, recent advancements in targeted therapy offer encouraging strategies to address the complexities associated with treatment of *NUP98*‐r AML.

Currently, various preclinical studies are exploring the unique properties of *NUP98*‐r AMLs, providing valuable insights into potential therapeutic strategies. These investigations suggest that FLT3, Menin, and BCL2 inhibitors, either alone or in combination with chemotherapy or hypomethylating agents, may offer therapeutic benefits for *NUP98*‐r AML patients. Encouraged by promising preclinical findings, these drugs and their combinations are now being evaluated in clinical trials that involve AML patients with *NUP98* rearrangements (Table 3).

## SUMMARY AND CONCLUSIONS

*NUP98* translocations are common genetic alterations in pediatric AML that are associated with dismal prognosis. *NUP98* fusions often co‐occur with a set of additional somatic mutations that confer a proliferative advantage to AML cells and add more complexity to the disease, demanding more effective therapeutics. Though novel insights into the molecular mechanism of NUP98 fusion protein‐driven leukemia have led to various new therapeutic opportunities, many questions remain. As most targeted AML agents have been studied and approved in adult AML, there is a critical need to establish their efficacy and pharmacokinetics specifically for pediatric patients. Thus, it is of vital importance to understand the molecular mechanisms underlying oncogenic transformation by NUP98 fusion oncoproteins which are prevalent in pediatric leukemia. Detailed analysis of individual samples with different *NUP98* fusions and comparison of their transcriptional landscape together with mechanistic investigations will open new and more efficient therapeutic avenues towards efficient treatments for patients suffering from these highly aggressive AMLs.

## AUTHOR CONTRIBUTIONS

Milad Rasouli and Selina Troester contributed equally as first authors. Olaf Heidenreich, Florian Grebien, Bianca F. Goemans, and C. Michel Zwaan contributed equally as last authors.

## CONFLICT OF INTEREST STATEMENT

Olaf Heidenreich received research funding from Syndax and Roche. The other authors declare no conflicts of interest.

### FUNDING

This work was supported by KiKa program grant 329 to Olaf Heidenreich. Work in the Grebien laboratory was supported by the Austrian Science Fund (FWF, grants no. TAI‐490 and P35628). Selina Troester is the recipient of a DOC fellowship of the Austrian Academy of Sciences at the University of Veterinary Medicine.

## Data Availability

Data sharing is not applicable to this article as no new data were created or analyzed in this study.
